# Imaging classification in videolaryngoscopy: are we on the right track?

**DOI:** 10.1016/j.bjane.2025.844671

**Published:** 2025-08-13

**Authors:** Daniel Perin, Mauricio Luiz Malito, Maurício do Amaral Neto

**Affiliations:** Centro de Treinamento em Vias Aéreas (CTVA), São Paulo, SP, Brazil

The perfect classification of the videolaryngoscopy (VL) image is the Holy Grail of modern intubation. It is important to classify and then stratify the risks of difficulty or failed intubation. Since the introduction of the Macintosh^1^ and Miller^2^ laryngoscope blades, airway management has evolved, especially in the last two decades.

Jack Pacey (a surgeon) developed in 2001 the first VL which achieved an indirect view of the glottis, permitting tracheal intubation independent of a direct line of sight.^3^ Levitan described the percentage of glottic opening (POGO) score using direct laryngoscopy, in 1999, with static images of the glottic opening. The method was proposed to replace the Cormack & Lehane (C&L) grades 1 and 2 with the POGO score.^4^

Prof. Cook described in 2000 a new classification of the original C&L adding a separation of the grades 2 and 3 in grade 2A, 2B, 3A and 3B. Nevertheless, introduced the concept of easy (grade 1 and 2A), restricted (grade 2B and 3A) and difficult (grade 3B and 4). Using this new classification, an easy view predicted easy intubation in 95% of the cases and difficult view was associated with difficult intubation in three-quarters of cases.^5^

Different anesthesia societies recommend the use of videolaryngoscopy for airway management and it is clear now that larynx view is enhanced with the device.^6^, ^7^, ^8^

The study entitled “VCI Spain: protocol for a prospective multicenter observational study to validate a standardized classification tool for tracheal intubation using videolaryngoscopy” from our Spain colleagues is well designed and aims to address the gap between C&L and POGO classifications. The score has three parts: Blade type (Macintosh (Mac) or Hyperangulated (HA)), POGO (0-25%, 50-75% and > 75%) and Intubation (Easy, Difficult and Failed).^9^

Several considerations should addressed when proposing a new classification. The first important decision is to define the best tool for a specific patient. How should be the initial approach to choose the blade based on phenotypes or clinical conditions?

This is fundamental to offer a better chance for the patient to be successfully intubated on the first attempt without complications. Is it time to abandon the options and suggest which blade should be used for the first attempt (Mac or HA)?

When the option is available, the decision-making process is more difficult and relies on user’s experience related to cognitive ease (system 1) or cognitive strain (system 2).^10^

Depending on which blade is chosen for the patient, an intubation can shift from difficult to easy, or vice versa. Also, the size of the blade (3 vs 4) can influence the POGO score and, consequently, the result of a successful intubation.^11^

The POGO classification is valuable, but it’s crucial to understand that the visualization proposed is at the exact moment, immediately before advancing the endotracheal tube through the glottis and not the initial or best view. The use of percentage is very good when analyzing a steady picture.

It is more complicated to be sure of the percentage of glottic opening when you are doing the intubation in real time. Relying on anatomical parameters seems to be more objective. Another point is that the POGO classification does not differentiate the C&L grade 3A (restricted) from the grades 3B and 4 (difficult). Are them clinically equivalent or, as proposed by Cook, they are different and the resolution tools to achieve tracheal intubation are not the same?

The authors propose a good categorization regarding the intubation: easy, difficult or failed. Once again, the possibility of intubating a patient depends on various factors and the success is a result of a good strategy, technical and non-technical skills and situational awareness. Consequently, if the initial strategy is to use an adjunct as the first option and the operator obtains success, is this patient going to be labeled as difficult intubation?

It is understandable that different models of videolaryngoscopes are the reality of the hospitals, and the experience of the users varies among preferences and availability. On the other hand, it becomes harder to compare channeled and non-channeled devices in the case of difficult or failed intubation. Each device has some advantages and also disadvantages, and it should be determined by a direct comparison to understand the incidence of difficulty or failure. Experience with one type of videolaryngoscope does not equate to skill with all videolaryngoscopes.^12^

Finaly, it is essential to refine the classification of the videolaryngoscopic images and translate it into clinical practice to augment the assertiveness of the intubation process and security for the patients. Nonetheless, it is also important to understand the difficulty to standardize a procedure involved with different perspectives and devices.

The VCISpain study^9^ will be a lighthouse to guide the next steps for new research in the field of image classification and standardization with videolaryngoscopes.

## Conflicts of interest

The authors declare no conflicts of interest.
